# Cerebrospinal fluid leaks following intradural spinal surgery—Risk factors and clinical management

**DOI:** 10.3389/fsurg.2022.959533

**Published:** 2022-09-20

**Authors:** Moritz Lenschow, Moritz Perrech, Sergej Telentschak, Niklas von Spreckelsen, Julia Pieczewski, Roland Goldbrunner, Volker Neuschmelting

**Affiliations:** Center for Neurosurgery, University Hospital of Cologne, Cologne, Germany

**Keywords:** cerebrospinal fluid leak, drainage, postoperative complications, mobilization, spine

## Abstract

**Background:**

Cerebrospinal fluid leakage (CSFL) following spinal durotomy can lead to severe sequelae. However, while several studies have investigated accidental spinal durotomies, the risk factors and influence of clinical management in planned durotomies remain unclear.

**Methods:**

We performed a retrospective analysis of all patients who underwent planned intradural spinal surgery at our institution between 2010 and 2020. Depending on the occurrence of a CSFL, patients were dichotomized and compared with respect to patient and case-related variables as well as dural closure technique, epidural drainage placement, and timing of mobilization.

**Results:**

A total of 351 patients were included. CSFL occurred in 4.8% of all cases. Surgical indication, tumor histology, location within the spine, previous intradural surgery, and medical comorbidities were not associated with an increased risk of CSFL development (all *p* > 0.1). Age [odds ratio (OR), 0.335; 95% confidence interval (CI), 0.105–1.066] and gender (OR, 0.350; 95% CI, 0.110–1.115) were not independently associated with CSFL development. There was no significant association between CSFL development and the dural closure technique (*p* = 0.251), timing of mobilization (*p* = 0.332), or placement of an epidural drainage (*p* = 0.321).

**Conclusion:**

CSFL following planned durotomy pose a relevant and quantifiable complication risk of surgery that should be factored in during preoperative patient counseling. Our data could not demonstrate superiority of any particular dural closure technique but support the safety of both early mobilization within 24 h postoperatively and epidural drainage with reduced or no force of suction.

## Introduction

Cerebrospinal fluid leakage (CSFL) is a severe complication following incidental or planned durotomy in spinal procedures. Possible sequelae include infectious complications ranging from wound infections to meningitis, intracranial hypotension and hemorrhage, nerve root compression syndromes, and back pain, as well as the necessity for revision surgery, increased morbidity, a prolonged hospital stay, and higher healthcare costs (1, 2). However, the majority of studies on secondary CSFL investigate the setting of incidental durotomy; hence, there are limited data regarding patient and case-related risk factors for CSFL following planned durotomy, i.e., for the resection of spinal intradural tumors (3, 4). Furthermore, the perioperative management following planned durotomies is highly variable. Different surgical techniques for dural closure are available, the benefit of epidural drainage placement remains unclear, and the utility of postoperative immobilization has been discussed controversially. Thus, we aimed to identify possible risk factors for the occurrence of CSFL following intradural spine surgery in order to improve preoperative patient counseling and to analyze perioperative management strategies.

## Materials and methods

This study is a single-center retrospective analysis of 351 consecutive cases of intradural spine surgery conducted at our institution between June 2010 and December 2020, in accordance with the local ethics committee guidelines.

### Inclusion and exclusion criteria

All patients who underwent surgery for an intradural spinal pathology at our institution between June 2010 and December 2020 were included in this study. All cases with incomplete data records and patients under the age of 18 years at the time of surgery were excluded, as well as craniocervical pathologies involving the posterior fossa, i.e., Chiari malformations were excluded.

### Data collection

A postoperative CSFL was defined as either cerebrospinal fluid (CSF) leakage through the operative wound (CSF fistula) or development of a pseudomeningocele refractory to conservative therapy and requiring revision surgery. The incidence of CSFL within the study cohort was recorded over an observational period of at least 3 months following intradural surgery.

Medical charts were reviewed for the following variables: age, gender, medical comorbidities (hypertension, coronary heart disease, diabetes mellitus type II, chronic obstructive pulmonary disease, history of smoking), obesity (defined as a body mass index > 30), and duration of hospital stay.

In two cases, two different spinal segments distant from each other were operated on during the same procedure and recorded as two separate cases.

Type of surgical indication, tumor histology, surgical approach, and previous intradural operations on the same level were analyzed. Tethered cord syndrome and different forms of spinal dysraphism were summarized as developmental malformations. Tumor histology was determined according to the current WHO classification in effect.

Spinal surgery location was classified as cervical, thoracic, or lumbosacral. In case of a junctional segment, classification was based on the upper vertebra (e.g., a C7/T1 procedure was classified as a cervical case).

### Surgical technique

The dural exposure was classified according to the surgical report (laminectomy, hemilaminectomy, or laminoplasty). All durotomies were performed either median or paramedian along the longitudinal axis of the dural sac. All meningiomas were resected without removal of the associated dura, and the dural insertion site was coagulated corresponding to Simpson Grade II.

Perioperative management in our study cohort was based on a case-by-case decision according to the intraoperative findings and the preference of the treating surgeon; there were no institutional protocols regarding dural closure technique, epidural drain placement, or timing of mobilization.

Surgical dural closure technique was categorized into the following: (1) standalone suture repair, (2) suture plus a liquid fibrin sealant (TISSUCOL Duo S Immuno®, Baxter International Inc.), (3) suture plus a patch sealant (TachoSil®, Takeda Pharma and Hemopatch®, Baxter International Inc.), (4) suture plus a liquid fibrin sealant and a patch sealant, or (5) suture plus another combination and/or material [e.g., muscle, fascia or a dura replacement material (DuraGen® Plus, Integra LifeSciences; Neuro-Patch®, B. Braun; GORE PRECLUDE® Pericardial Membrane, W. L. Gore / Associates, Inc.].

The additional placement of an epidural drainage as well as the applied force of suction (full, reduced, none) were recorded. The full force of suction applied by the used drainage system was 150 mbar (150 hPa).

The ordered duration of postoperative strict bed rest was summed into an early mobilization (within 24 h) and a late mobilization group (after 24 h).

Procedure-related complications were classified as wound healing disorders without CSFL, epidural hemorrhage, neurological complications (postoperative new or deteriorated deficit, meningitis), urinary tract infections, pneumonia, or miscellaneous.

### Statistical analysis

Patients were dichotomized according to the occurrence of CSFL. Differences between the two groups were analyzed using descriptive statistics. To compare categorical variables, the Chi-square and Fisher's exact tests were used, when appropriate. Continuous variables were tested for normality using the Kolmogorov–Smirnov test. Group means with normally distributed data were compared using the two-sided unpaired Student's *t* test and the Mann–Whitney *U* test in case of non-normally distributed data. Continuous variables are reported as mean and standard deviation (mean ± SD) or as median and range (minimum, maximum). Risk is reported as absolute risk, unless otherwise stated. All calculations were performed using SPSS software (Version 27, IBM SPSS Statistics for Windows, Armonk, NY, USA). A *p*-value <0.05 was considered as statistically significant.

## Results

### Patient characteristics

A total of 351 patients who underwent surgery for an intradural spinal pathology were included. Median patient age was 55 years (range: 19–94), and 50.1% were female. The most common surgical indication was resection of an intradural tumor (75.5%). Of these, meningiomas (30.9%), intramedullary tumors (30.2%), and nerve sheath tumors (28.3%) were the most frequent histological diagnoses. The most common surgical site was the thoracic spine (49.9%), followed by lumbosacral (33.3%) and cervical spine (16.8%). In 13.1%, intradural surgery had previously been performed at the same level. Detailed patient characteristics including medical comorbidities are displayed in Table 1.

**Table 1:** Patient and case-related factors.

	No CSFL	CSFL	*p*-value
	*n* = 334	*n* = 17	
	N (row %)	N (row %)	
Age (median, range)	55 (19-94) years	50 (21-72) years	0.049
≥55 years	159 (92.4%)	13 (7.6%)	0.025
<55 years	175 (97.8%)	4 (2.2%)	
Sex			
Female	172 (97.7%)	4 (2.3%)	0.027
Male	162 (92.6%)	13 (7.4%)	
Surgical indication			
Intradural tumor	255 (95.1%)	13 (4.9%)	0.991
Arachnoid cyst	25 (92.6%)	2 (7.4%)	0.630
Arachnoiditis	12 (100.0%)	0 (0.0%)	1.000
Developmental malformations	21 (91.3%)	2 (8.7%)	1.000
Miscellaneous	21 (100%)	0 (0.0%)	0.655
Tumor histology			
Meningioma	78 (95.1%)	4 (4.9%)	0.987
Nerve sheath tumors	72 (97.3%)	2 (2.7%)	0.389
Intramedullary tumor	65 (94.2%)	4 (5.8%)	0.754
Intradural metastatic tumors	12 (92.3%)	1 (7.7%)	0.482
Miscellaneous	28 (93.3%)	2 (6.7%)	0.616
Location			
Cervical	59 (100%)	0 (0.0%)	0.088
Thoracic	166 (94.9%)	9 (5.1%)	0.810
Lumbosacral	109 (93.2%)	8 (6.8%)	0.291
Previous intradural surgery at the same level			
Yes	52 (91.2%)	5 (8.8%)	0.131
No	282 (95.9%)	12 (4.1%)	
Hypertension			
Yes	105 (95.5%)	5 (4.5%)	0.861
No	229 (95.0%)	12 (5.0%)	
Coronary heart disease			
Yes	19 (100%)	0 (0.0%)	0.612
No	315 (94.9%)	17 (5.1%)	
Diabetes mellitus			
Yes	31 (96.9%)	1 (3.1%)	1.000
No	303 (95.0%)	16 (5.0%)	
Chronic obstructive pulmonary disease			
Yes	11 (91.7%)	1 (8.3%)	0.454
No	323 (95.3%)	16 (4.7%)	
Smoking			
Yes	35 (89.7%)	4 (10.3%)	0.107
No	299 (95.8%)	13 (4.2%)	
Obesity			
Yes	55 (96.5%)	2 (3.5%)	0.839
No	234 (94.7%)	13 (5.3%)	
Not available	45	2	

CSFL, cerebrospinal fluid leakage.

### Perioperative factors

Surgical exposure mainly involved a single level (82.9%) and the majority of cases were operated *via* a hemilaminectomy (46.2%) or laminoplasty (21.9%). Dural closure was performed using a combination of suture plus a sealant patch in 72.9%, suture plus a liquid sealant as well as a sealant patch in 10.8%, suture plus other materials (e.g. fascia or muscle) in 12.3%, suture plus a liquid sealant in 1.4%, and dural suture was not augmented in 2.6% of all cases (Table 2). An epidural drainage was placed in 191 (54.4%) cases. Of those, reduced suction was applied in 117 (61.3%) and no suction in 74 (38.7%) cases; full suction was never applied. Mean duration of bed rest was 1.6 (±1.3) days. Of all patients, 53.0% were mobilized within 24 h and 47.0% after 24 h (Table 3).

**Table 2:** Intraoperative factors.

	No CSFL	CSFL	*p*-value
	*n* = 334	*n* = 17	
	N (row %)	N (row %)	
Number of exposed levels			
One level	275 (94.5%)	16 (5.5%)	0.506
Two levels	33 (100%)	0 (0.0%)	0.667
Three or more levels	26 (96.3%)	1 (3.7%)	0.630
Choice of approach			
Laminoplasty	74 (96.1%)	3 (3.9%)	0.774
Hemilaminectomy	154 (95.1%)	8 (4.9%)	0.939
Laminectomy	41 (95.3%)	2 (4.7%)	1.000
Interlaminar access	46 (95.8%)	2 (4.2%)	1.000
Other	17 (89.5%)	2 (10.5%)	0.233
Corpectomy	2 (100%)	0 (0.0%)	1.000
Dural closure technique			
Suture only	9 (100%)	0 (0.0%)	1.000
Suture + liquid sealant	4 (80.0%)	1 (20.0%)	0.221
Suture + patch sealant	245 (95.7%)	11 (4.3%)	0.413
Suture + liquid and patch sealant	37 (97.3%)	1 (2.6%)	1.000
Other	39 (90.7%)	4 (9.3%)	0.141

CSFL, cerebrospinal fluid leakage.

**Table 3:** Perioperative factors.

	No CSFL	CSFL	*p*-value
	*n* = 334	*n* = 17	
	N (row %)	N (row %)	
Drainage insertion			
Yes	184 (96.4%)	7 (3.7%)	0.321
No	150 (93.8%)	10 (6.3%	
Force of suction			
Full	0 (0.0%)	0 (0.0%)	0.956
Reduced	113 (96.6%)	4 (3.4%)	
None	71 (95.9%)	3 (4.1%)	
Bed rest (mean, standard deviation)	1.5 ± 1.3 days	2.0 ± 1.2 days	0.121
Timing of mobilization			
Early mobilization (<24 h)	179 (96.2%)	7 (3.8%)	0.332
Late mobilization (>24 h)	155 (93.9%)	10 (6.1%)	

CSFL, cerebrospinal fluid leakage.

### Risk factors for cerebrospinal fluid leak

**Figure 1:**
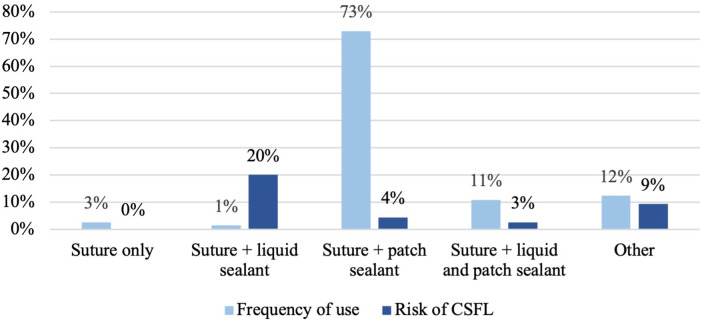
Dural closure technique and risk of cerebrospinal fluid leak development.

CSFL occurred in 17 cases (4.8%). In univariate analysis, age (median 55 vs. 50 years, *p* = 0.025) and gender (2.3% female vs. 7.4% male, *p* = 0.027) were significantly associated with CSFL development, but neither age [odds ratio (OR), 0.335; 95% confidence interval (CI), 0.105–1.066] nor gender (OR, 0.350; 95% CI, 0.110–1.115) remained as independent risk factors in multivariate analysis (Table 4). The following disease, surgery, and patient-related variables were tested as potential cofactors and found not to influence the risk of CSFL development: surgical indication (all *p* > 0.1), tumor histology (all *p* > 0.1), location (all *p* > 0.05), previous intradural surgery (*p* = 0.131), medical comorbidities (all *p* > 0.1), number of exposed levels (all *p* > 0.1), choice of approach (all *p* > 0.1), dural closure technique (all *p* > 0.1, Figure 1), drainage insertion (*p* = 0.321), force of suction (*p* = 0.537), and timing of mobilization (*p* = 0.332).

**Table 4:** Binary logistic regression analysis.

Factor	Effect	Odds ratio (95% CI)	*p*-value
Age	< 55vs. >55 years	0.335 (0.105–1.066)	0.064
Gender	Male vs. female	0.350 (0.110–1.115)	0.076

CI, confidence interval.

### Complications following early and late mobilization

Wound healing disorders occurred in 6 cases (1.6%), epidural hematoma in 8 cases (2.3%), urinary tract infection in 12 cases (3.4%), pneumonia in 1 case (0.3%), and new neurological deficits in 26 cases (7.4%, Table 5). The incidence of urinary tract infections was significantly higher in the early mobilization group (5.4% vs. 1.2%; *p* = 0.039), and the two groups did not differ with respect to other complications (all *p* > 0.1).

**Table 5:** Complications following early and late mobilization.

	Early mobilization	Late mobilization	*p*-value
	*n* = 186	*n* = 165	
	N (column %)	N (column %)	
Neurological deficit	18 (9.7%)	8 (4.8%)	0.103
Wound healing deficits	5 (2.7%)	1 (0.6%)	0.220
Epidural bleeding	3 (1.6%)	5 (3.0%)	0.482
Urinary tract infections	10 (5.4%)	2 (1.2%)	0.039
Pneumonia	1 (0.5%)	0 (0.0%)	1.000
Miscellaneous	9 (4.8%)	3 (1.8%)	0.148

## Discussion

In this series, we report on 351 planned durotomies in adult patients with regard to factors influencing CSFL, making this the second largest series in this regard and the largest to investigate the impact of postoperative mobilization and epidural drainage placement as well as the first to investigate medical comorbidities.

The overall risk of CSFL development was 4.8%, which is in line with previous studies reporting an average risk ranging from 0 to 10% (3–7).

In general, reports regarding risk factors for CSFL following planned durotomies are sparse. Two studies investigating the impact of age and gender on CSFL development found no association, which is in line with the findings of our study. Regarding the impact of location, our study is consistent with previous reports that did not show a significant correlation (6, 8). Of note, no CSFL occurred in the cervical spine in our cohort, possibly attributed to the fact that merely 17% of all intradural surgeries were performed in the cervical spine in our cohort in the first place. Contrary to reports on incidental durotomies, prior epidural surgery does not appear to be a risk factor for CSFL in case of planned durotomies (3, 5, 9, 10).

As this is the first to examine the impact of medical comorbidities on CSFL in planned durotomies, our study first provides evidence of no significant correlations in this regard.

While the choice of surgical approach and degree of dural exposure and bone removal was not found to influence the risk for CSFL, which is in line with previous findings (6), there was no dedicated analysis comparing open and minimally invasive approaches in our study due to the small number of patients being treated with minimally invasive approaches. However, minimally invasive techniques may offer advantages over open procedures in terms of reduced perioperative complications, including CSFL, for both accidental and planned durotomies (11–13).

Although additional (liquid or patch) sealants are frequently used to reinforce dural suture (97.4% in our study), their benefit remains uncertain (11).

Use of fibrin sealants has been discouraged by several authors following both planned and unplanned durotomies due to a lack of effectiveness in preventing CSFL (10, 14–16), even though two studies reported favorable results (4, 17). The effectiveness and the necessity of patch sealants to prevent CSFL are not sufficiently studied in planned durotomies. Favorable results were reported by Montano et al. who reported no CSFL requiring revision surgery after dural closure with TachoSil® in 35 intradural procedures (18). In contrast, other studies investigating patch sealants found no clinical benefit in terms of CSFL risk reduction, thus questioning their application (3, 6, 11). Overall, further research is warranted to investigate the utility of sealants in planned durotomies. Based on the currently available data as well as our findings, the use of additional sealants in case of adequate dural closure by suture may not provide further benefit.

The placement of an epidural drainage following durotomy is controversially discussed (16, 19). Our data showed no effect on CSFL development, suggesting a generally safe applicability with reduced or no force of suction. It does not allow further conclusions such as previous reports advocating for epidural drainage placement in order to reduce the risk of CSFL by influencing epidural pressure gradients and thus supporting wound healing (16, 20). Furthermore, our results are inconsistent with reports of increased complication rates, including CSFL, following epidural drainage in both planned and accidental durotomy (3, 21–24).

Bed rest following spinal durotomy remains a common measure, although in case of accidental durotomy, studies failed to show any benefit in terms of CSFL reduction. Accordingly, our series of planned durotomies showed no impact of early or late mobilization on the development of CSFL, consistent with the only other study currently available on this topic (4). Consequently, early mobilization after planned durotomy appears to be beneficial, as it is associated with a reduction in medical complications such as ileus, pneumonia, and deep vein thrombosis, as well as a reduced socioeconomic financial burden due to overall shorter hospital stays (25, 26). Of note, the overall complication rate did not differ significantly between the early and late mobilization groups in our study, possibly due to the small number of patients immobilized for more than three days in our cohort. Accordingly, we attribute the higher rate of urinary tract infections in the early mobilization group to the retrospective study design.

This study carries several limitations. First, our study is limited by its retrospective design and single-center patient population. Second, the risk of CSFL development might be underestimated due to the rather strict definition of a CSFL used in this study. CSFL was defined as cerebrospinal fluid leakage through the operative wound refractory to conservative therapy and necessitating operative revision surgery, which is in contrast with other publications that included conservatively managed cases as well. Third, the technique of dural closure, epidural drain placement, and timing of mobilization were not based on standardized protocols but at the discretion of the treating surgeon, potentially causing a selection bias. Finally, case numbers for certain techniques of dural closure were small, limiting statistical power.

## Conclusion

CSFL following planned durotomy pose a relevant complication risk of surgery that can be quantified for preoperative patient-specific counseling and consent. To minimize the risk of CSFL, our data highlight the importance of adequate dural closure by suture, and the use of additional sealants remains optional. In contrast to previous treatment recommendations, our results indicate the safety of both epidural drainage with reduced or no force of suction and early mobilization within 24 h following intradural surgery to prevent further complications.

## Data Availability

The raw data supporting the conclusions of this article will be made available by the authors, without undue reservation.
